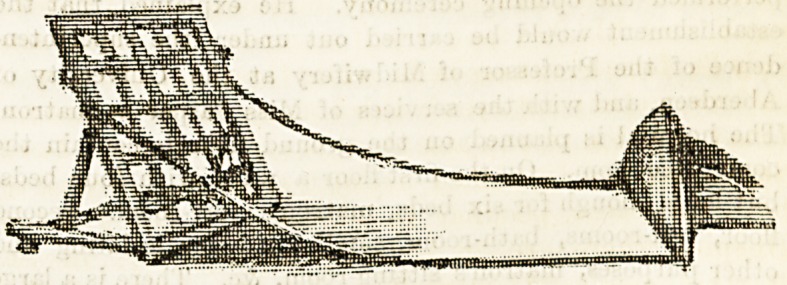# Practical Departments

**Published:** 1894-04-21

**Authors:** 


### PRACTICAL DEPARTMENTS.

### A COMFORTABLE CRUTCH.

The crutch in question has been commented upon before in the pages of The Hospital, but the inventor, Mr. Beaumont, has lately improved upon the original idea in such a manner as to make it of increased practical use. The drawing given below shows that the special feature of this crutch is its side handle, which enables the patient to support himself at once with a firmer grip and with less fatigue than can be the case with the ordinary crutch stick. It also far easier for the crutch to be kept in a perpendicular position. This handle has now been so contrived by means of a "side clip," that it can be moved up and down to suit various requirements in the matter of height, and it can be instantly fitted upon any crutch stick. Formerly it was fixed with a screw.

These recommendations undoubtedly place the crutch above those in general use amongst hospital patients, for whose benefit Mr. Beaumont's invention is largely intended, for the price is very small, two shillings a pair. The foot of the crutch may be shod with indiarubber or leather in order to prevent slipping. For those who may be obliged to make use of the help of such support the Beaumont crutch will certainly prove a comfort, and help to make locomotion under these circumstances less tedious and tiring than it would otherwise be.

### COMBINED BACK AND FOOT REST.

A practical difficulty is often experienced in the endeavour to comfortably support helpless patients with the ordinary bed rest. The tendency of the rest itself is to push the patient forward, and this gives a feeling of slipping and insecurity which militates largely against the comfort of such a support. The sketch here given is not that of a new invention, but one which it will be seen at once adds very greatly to the comfort of a sick person, and which is too often overlooked. Nurses know that these small-seeming points are large in importance when considered from the patient's point of view, but it is probably the exception rather than the rule to find the comfort-giving foot rest used in conjunction with the back rest in hospitals or elsewhere.

The strip of stout webbing which connects the two is provided with a buttonhole to attach it firmly to the side of the back rest, and it is a good plan to have the strip sufficiently long to enable it to be fastened, if desired, to the head-rail of the bed itself. It can then be used independently, in many ways an advantage.

For spinal cases it is particularly helpful, as with these patients there is a special tendency for the toes to fall forward in bed, and the rest, by keeping the feet in an upright position, acts as a counteracting agent. With the bed rest here shown the foot support is also sold, but the latter can, of course, be used with any form of back rest most preferred. The foot piece is well padded.

## Figures and Tables

**Figure f1:**
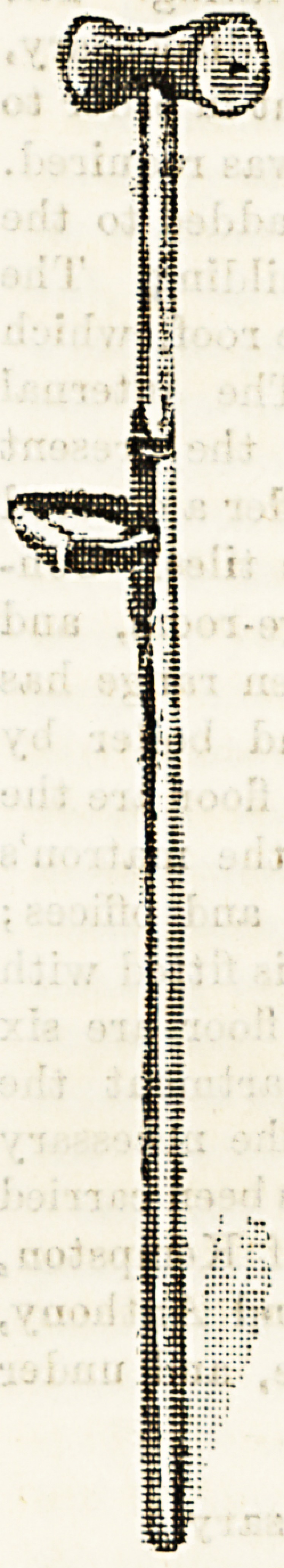


**Figure f2:**